# Nonsuicidal self-injury in sexual minority college students: a test of theoretical integration

**DOI:** 10.1186/s13034-015-0050-y

**Published:** 2015-07-08

**Authors:** Jennifer J. Muehlenkamp, Lori M. Hilt, Peter P. Ehlinger, Taylor McMillan

**Affiliations:** Department of Psychology, University of Wisconsin-Eau Claire, 105 Garfield Ave, Eau Claire, WI 54702 USA; Department of Psychology, Lawrence University, 347 Briggs Hall, 711 E. Boldt Way, Appleton, WI 54911 USA; Department of Psychiatry, Massachusetts General Hospital, Boston, MA 02114 USA; Eastern Illinois University, 600 Lincoln Ave, Charleston, IL 61920 USA

**Keywords:** Sexual minority, Youth, NSSI, Suicide, Self-injury, Self-harm, College, Minority stress, Burdensomeness

## Abstract

**Background:**

Individuals identifying as a sexual minority report engaging in nonsuicidal self-injury (NSSI) at substantially higher rates compared to their heterosexual peers. Given that NSSI is a known risk factor for suicide, it is important to understand the processes unique to being a sexual minority that increases risk for NSSI so that adequate prevention efforts can be established. The current study integrated Minority Stress Theory and the Interpersonal Theory of Suicide to test a model of NSSI and suicide risk.

**Methods:**

A total of 137 college students who identified as a sexual minority completed an anonymous on-line study assessing NSSI, suicidal thoughts/behaviors, and constructs of the minority stress and interpersonal theories. Two linear regressions using bootstrapping analyses were conducted to test our hypotheses.

**Results:**

Minority stress was directly associated with NSSI and via perceived burdensomeness, explaining 27 % of the variance. NSSI was associated with increased risk for suicide thoughts/behaviors directly, and through acquired capability, explaining 45 % of the variance.

**Conclusions:**

These findings provide evidence that unique stressors individuals face as a result of their sexual minority status increases risk for self-harm by influencing cognitive and emotional processes such as burdensomeness and acquired capability. Implications for prevention, intervention, and future research are briefly discussed.

Adolescents and young adults represent developmental age groups with particularly high rates of both non-suicidal self-injury (NSSI) and suicidal behaviors [1, 2]. However, studies consistently find that individuals who identify as gay, lesbian, bisexual, transgendered, or gender-queer (LGBTQ; sexual minority) report notably higher rates of psychiatric symptoms, suicidal ideation and attempts, and NSSI relative to their heterosexual peers [3–6]. Within the general population of adolescents and college students, sexual minority individuals are two to seven times more likely to attempt suicide [7] and approximately three to five times more likely to engage in NSSI [8, 9]. Additionally, Dickey and colleagues [10] reported that even among transgender adults, a greater proportion of those who identified with a non-heterosexual orientation reported engaging in NSSI relative to those identifying as heterosexual. These findings support other data suggesting that being a member of a sexual minority group confers risk for both NSSI and suicide [11]. Compounding this concern is evidence documenting that a history of NSSI is associated with an 11-fold increased risk for suicide among sexual minority youth [9]. Understanding the factors that influence the development of NSSI within sexual minorities is essential to advancing evidence-based prevention and intervention models.

While current research on NSSI has documented the high prevalence of self-injury among sexual minorities, it has largely failed to explain *why* rates are so high. Minority stress theory [12] may offer a valid explanation for the elevated rates of NSSI observed within this population. Individuals who identify as LGBTQ often experience disproportionate social stressors such as prejudice, individual and institutional discrimination, victimization, and family rejection [13–15] because of their minority status. It is these negative social experiences, along with the expectation that prejudicial events will continue to occur, that are believed to contribute to the increased mental health difficulties observed among sexual minorities [12]. Research consistently finds that sexual minorities report significantly higher rates of harassment, discrimination, and violence relative to heterosexuals [16, 17]; all of which are uniquely associated with lower self-esteem and increased depression, anxiety, suicidal ideation, and NSSI [17–20]. So while sexual minority individuals may share many of the same risk factors for NSSI as heterosexual individuals (e.g., mental illness, emotion dysregulation, impulsivity), they also face unique social stressors that can compound their risk simply by being a member of a minority group.

Furthermore, Hatzenbuehler [21] points out that experiences of minority stress can increase risk for mental health problems by altering cognitive and interpersonal processes. For example, frequent experiences of rejection or victimization are likely to contribute to feelings of isolation, alienation, and low self-esteem which are primers for symptoms of depression. Repeated experiences of discrimination and/or violence are likely to fuel expectations of future rejection which may contribute to feelings of anxiety and hinder interpersonal interactions. A recent study including a national sample of college students found that sexual minority students reported more socially based stressors than their heterosexual peers, and experiencing social stressors, along with victimization, was associated with a higher odds of reporting NSSI [16]. Research also notes that a large portion of sexual minority individuals are resilient and do not suffer from significant mental distress, engage in NSSI, or become suicidal [22]. Individuals who show high levels of resilience and mental health appear to experience strong social support from friends and family [9, 20, 23] as well as live, or go to school, in gay-friendly environments [24, 25]. Thus, it appears that negative interpersonal events experienced due to sexual minority status is associated with greater NSSI, and that a complex set of processes or variables are accounting for the associations observed between minority stress and NSSI.

Originally created to explain suicide risk, Joiner’s Interpersonal Theory of suicide [26, 27] has demonstrated applicability to NSSI [28] and may help explain some of the processes linking minority stress to NSSI. The Interpersonal Theory posits that individuals come to desire suicide and increase their propensity for self-harm behavior when they perceive themselves to be a significant burden on, or liability to, others (i.e., perceived burdensomeness) and experience a sense of thwarted belongingness (e.g., sense of loneliness, not fitting in). If these states of burdensomeness and low belonging are perceived as enduring and unchanging, risk for suicide is enhanced. These ideas may also help to explain the emergence of NSSI among sexual minority youth who have experiences of social exclusion. Being discriminated against or shunned by one’s peers because of sexual minority status can lead to especially strong feelings of being unworthy, lonely, and rejected [25, 29]. The experiences of discrimination, victimization/bullying, and expectations of social rejection may increase risk for NSSI within sexual minorities through the association to thwarted belongingness.

Sexual minority individuals may also view themselves as a burden on those they care about due to societal designation of non-heterosexuals as low social status [30, 31], and their inability to meet certain social standards or expectations of family (e.g., right to marry; perceive sexual orientation as distressing to parents). Perceptions of burdensomeness may be further exacerbated if an individual believes their sexual minority status brings heightened stress, shame, negative experiences, or expectations for advocacy and defense to their supportive family and friends [32]. Perceptions of being a burden may be especially important to youth as their social support and sense of connection is still rooted with the family unit. Very few studies exist examining perceived burdensomeness in NSSI, but one study found that sexual minority college students reported significantly higher perceived burdensomeness relative to their heterosexual peers, and burdensomeness was significantly correlated with NSSI [33]. The limited data indicates it is plausible that sexual minority status is related to NSSI because of its connection to perceived burdensomeness.

Joiner [26] specifies a third construct, acquired capability, as the final element within his theory to understanding why individuals who desire suicide take action. Acquired capability is best understood as a habituation to fear and pain that can result from repeated exposure to painful situations. NSSI is viewed as a behavior that contributes to, and builds, a person’s acquired capability and therefore, represents a risk factor for suicide attempts [28]. Within a variety of samples, NSSI has been identified as a significant predictor of future suicide behavior even after accounting for other known suicide risk factors such as depression, hopelessness, suicidal ideation, and past suicide attempts [34, 35]. However, the direct effect of NSSI on suicide is notably stronger within sexual minority youth relative to heterosexual youth [9]. These data underscore the salient role of NSSI for suicide risk but, fail to offer an explanation of the processes for how NSSI confers this substantial increase in risk. NSSI is likely to exacerbate an individual’s acquired capability for suicide, and this risk may be exponentially greater for sexual minority youth who may already have elevated acquired capability because of their increased exposure to physical violence and hate crimes related to sexual orientation [14].

The elevated rates of NSSI and suicide among sexual minority youth highlights the need for careful study of the factors unique to the sexual minority experience that heighten risk. However, tests of theoretical models explaining the emergence of NSSI within this high risk population, as well as the robust connection between NSSI and suicide in sexual minority youth are lacking. The purpose of the current study was to integrate Minority Stress theory with the Interpersonal Theory of suicide in an effort to explore the processes influencing NSSI and suicide risk among sexual minority college students. We hypothesized two mediational models. First, we hypothesized that the positive relationship between sexual minority stress and NSSI would be mediated by both perceived burdensomeness and thwarted belongingness. Second, we hypothesized that the positive relationship between NSSI and suicide would be mediated by acquired capability.

## Method

### Participants

Participants included 137 college students who were at least 18 years old (M = 19.86, SD = 1.65), identified as a sexual minority (e.g., lesbian, gay, bisexual, transgender, queer, questioning), and completed our online survey. They were recruited primarily through emails sent to LGBTQ advocacy groups on the campuses of the first two authors and via snowball sampling. The survey was also posted on the psychology department’s research subject pool management system affiliated with the first author.

Participants were asked about their gender and sexual identities and were instructed to check any that apply. They identified as female (74 %), male (16 %), transgender (3 %), intersex or gender fluid (3 %), and cisgender (37 %). Participants also identified as gay (13 %), lesbian (18 %), asexual (4 %), bisexual (29 %), questioning (24 %), pansexual (15 %), queer (17 %), and demisexual (4 %). Although some participants identified as heterosexual (5 %), they were included if they also identified as a sexual minority. Participants indicated their racial/ethnic identification (multiple answers allowed) with most identifying as White (89 %), followed by Asian (6 %), multi-racial (5 %), Black (4 %), American Indian/Alaska Native (3 %), Hmong (1.5 %), Hispanic/Latino (1.5 %), and Pacific Islander (1 %). Most participants reported being full-time students (94.2 %), living on campus (63.5 %), and living with a roommate (64.2 %). Participants were eligible to enter a drawing for one of four 50-dollar gift cards as compensation for their participation in the study. Students completing the study through the psychology subject pool also received two research participation credits for their courses.

### Procedure

After approval from both affiliated University’s IRBs, participants were invited through email to complete an online study examining “experiences and well-being of LGBTQ individuals”. Participants were able to complete the survey anywhere they had uninterrupted internet access for 20–30 min. Data were collected anonymously using Qualtrics, an academic online survey platform, and IP tracking functions were disabled. Informed consent was obtained and participants certified their age and enrollment status at a university in order to proceed to the survey material. After completing demographic questions, participants were presented with each questionnaire in a randomized order. On completion of the study, participants were presented with a debriefing screen thanking them for their involvement in the study. After debriefing, if participants desired to be entered in the gift card drawing, a link was provided that redirected participants to a new survey so that their entry in the drawing was not connected with their anonymous survey responses.

### Measures

#### Minority stress

We used the Schedule of Sexually Discriminatory Events (SSDE; House, Coppeans, & Stepleman: *The Schedule of Sexually Discriminatory Events,* unpublished) to measure the experience of discriminatory events related to sexual orientation. The SSDE has 19 items that were modified from the Schedule of Racist Events [36] so that the items reflected discriminatory experiences that sexual minorities may encounter. Example items are: “How many times have you been treated unfairly by teachers and professors because you are a sexual minority?” and “How many times have you been called an offensive name because you are a sexual minority?” Internal consistency in this study was excellent (α = .91).

To measure expectation of rejection, we used a self-designed series of questions asking participants about their degree of worry related to expecting negative interactions (e.g., “I worry that I will be criticized because of my sexual orientation or gender expression/identity”; “I worry that I will be discriminated against in a job interview because of my sexual orientation or gender expression/identity”). Internal consistency was good (α = .85).

We combined discriminatory events and expectations of rejection into a single minority stress variable in order to test the first hypothesis. This was done by creating z-scores for each scale and then taking the average.

#### Interpersonal theory

The Interpersonal Needs Questionnaire (INQ; [37]) consists of fifteen items designed to measure the extent to which participants feel like a burden on other people in their lives (e.g., “These days the people in my life would be better off if I were gone”), and the extent participants feel disconnected from others (e.g., “These days I feel disconnected from other people”). Nine items comprise the thwarted belongingness scale and six items assess perceived burdensomeness. Participants rate the degree to which each item is true for them using a seven-point Likert scale (1 = not at all true for me, 7 = very true for me). Response values within each subscale are summed (and then averaged) so that higher scores indicate higher levels of thwarted belongingness and perceived burdensomeness. Internal consistency was good for both the burdensomeness subscale (α = .70), and the belongingness subscale (α = .82).

To measure acquired capability, we used the Acquired Capability Scale [38]. This study utilized the 20-item version of the scale, which is the most widely used [39]. The scale is designed as a continuous construct, measuring both fearlessness (e.g., “Things that scare most people do not scare me”), and increased pain tolerance (e.g., “The pain involved in dying frightens me”). Items are rated on a five point Likert scale (0 = “Not at all like me”, to 4 = “Very much like me”), and summed to create a total score where higher scores indicate a higher acquired capability. Internal consistency in the current study was high (α = .85).

#### NSSI and suicide behavior

To assess NSSI a behaviorally descriptive scale based upon the Inventory of Statements About Self-Injury [40] was used. The assessment of NSSI consisted of seven self-report items that asked participants to report the lifetime frequency of engaging in specific behaviors of self-injury without suicidal intent (e.g., cutting/carving, burning, self-battery/ banging). A continuous NSSI score was calculated by summing the frequency responses for each of the items. Suicide behavior was assessed with a single question from the Suicidal Behaviors Questionnaire (SBQ-R; [41]), “*Have you ever seriously thought about or attempted to kill yourself*?” Participants responded using a 6-point scale so that higher values corresponded with more serious suicidal thoughts and behavior: 1 = never, 2 = passing thought, 3 = plan without intent, 4 = plan with intent, 5 = attempt without serious intent, 6 = attempt with serious intent.

## Results

Over half of the sample reported lifetime NSSI (62.8 %) as well as suicidal thoughts or behaviors (66.4 %); 42.9 % of the sample reported experiencing both NSSI and suicidal thoughts/behavior. Means, standard deviations, and bivariate correlations for all variables are presented in Table 1. NSSI was significantly positively correlated with all the variables. Suicide thoughts/behaviors were significantly correlated with all the variables except belongingness.

**Table 1:** Means, standard deviations, and bivariate correlations among study variables

	1	2	3	4	5	6
1. Minority stress	--					
2. Thwarted belongingness	.11	--				
3. Burdensomeness	.20*	.71**	--			
4. Acquired capability	.07	.03	.14	--		
5. NSSI	.39**	.21*	.41**	.37**	--	
6. Suicide thoughts/behaviors	.31**	.14	.38**	.45**	.63**	--
Means	.35	3.34	2.74	59.32	8.02	2.55
SDs	.75	1.16	0.96	13.57	9.60	1.61

Note. *p < .05; **p < .01

Linear regression analyses were used to test our study hypotheses. We ran our regressions using PROCESS for SPSS [42]. This procedure uses bootstrapping analyses to estimate 95 % bias corrected confidence intervals (1000 resamples) to assess both direct and indirect effects.

**Fig. 1:**
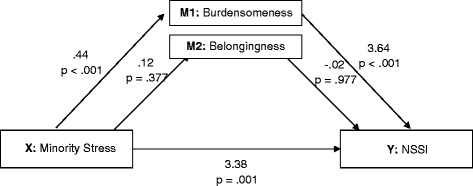
Results of a meditational model showing associations among minority stress, the proposed mediators (perceived burdensomeness and thwarted belongingness) and NSSI

*Hypothesis 1*: To examine whether minority stress predicted NSSI via the mediating role of perceived burdensomeness and thwarted belongingness, we specified a model with two mediators, *F*(3,133) = 16.31, *p* < .001, *R*^2^ = .27. The direct effect of minority stress on NSSI was significant, *t* = 3.34, *p* < .001 (CI = 1.81 – 5.78). The indirect effect through perceived burdensomeness was also significant (CI = .69 – 2.83), but not through thwarted belongingness (CI = −.30 - .20). See Fig. 1.

**Fig. 2:**
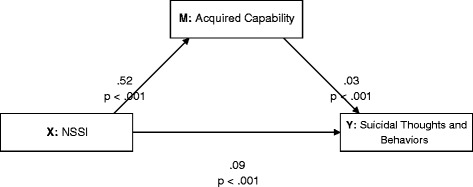
Results of a mediational model showing associations among NSSI, the proposed mediating variable (acquired capability) and suicidal thoughts and behaviors

*Hypothesis 2*: To examine whether NSSI predicted suicidal thoughts and behaviors via acquired capability, we specified a single mediator model, *F*(2,134) = 55.77, *p* < .001, *R*^2^ = .45. The direct effect of NSSI on suicidal thoughts and behaviors was significant, *t* = 7.90, p < .001 (CI = .07 - .12). The indirect effect through acquired capability was also significant (CI = .01 - .03); see Fig. 2.

## Discussion

This study of sexual minority college students demonstrated that the high rates of NSSI and suicide behavior can be explained by integrating Minority Stress Theory with the Interpersonal Theory of suicide. We found support for our first hypothesis with results showing that minority stress predicted NSSI via the indirect effect of perceived burdensomeness. Our second hypothesis was also supported, with results indicating that NSSI was associated with suicide behavior via the indirect effect of acquired capability. Understanding the mechanisms of increased risk for self-injury among sexual minority college students has important implications for prevention and intervention efforts.

Consistent with other studies examining the prevalence of NSSI within sexual minority youth, a large portion of our sample endorsed self-injury. Prior research has documented high rates of NSSI among sexual minority youth relative to heterosexual peers [4, 9, 43]. While our current data did not compare sexual minority and heterosexual youth, our findings are consistent with the existing research suggesting that identifying with a sexual minority status confers increased risk for NSSI [44]. In addition, a concerning proportion of participants in our sample endorsed having suicidal thoughts and behaviors, and engaging in NSSI was associated with increased suicidal thoughts and/or behavior. Despite recent reviews documenting that a majority of sexual minority youth are well adjusted and have low risk for NSSI or suicide [22], the current results underscore the need to monitor and assess for possible NSSI and suicidal thinking among sexual minority youth.

One explanation for the elevated rates of NSSI observed within sexual minority youth is that the unique stressors associated with their minority status contribute to psychosocial processes that are known to increase mental health problems, such as a sense of social alienation and rejection. Findings from the current study are consistent with this notion, as minority stress, thwarted belongingness, and perceived burdensomeness were all positively associated with NSSI. Furthermore, minority stress retained a significant direct effect on NSSI even when mediators were included in our model, lending credibility to minority stress theory for understanding NSSI risk. It appears that experiencing elevated societal stressors as a result of minority status may overwhelm coping skills, particularly if compensatory resilience resources are unavailable [45]; thereby, contributing to NSSI. Minority stress theory also suggests that being repeatedly exposed to hegemonic societal values and stigma related to sexual minority status contributes to internalized homonegativity [12], which is then related to low self-esteem and self-deprecation [15, 46]. One of the more common reasons given for why people engage in NSSI is to self-punish, or to help regulate self-hate/self-deprecation [40, 47, 48]. Thus, some sexual minority individuals may turn to NSSI to help regulate strong feelings of internalized homonegativity. Additional studies are needed to test this hypothesis.

Along with having a direct effect on NSSI, minority stress showed an indirect effect through perceived burdensomeness. This finding supports Hatzenbuehler’s [21] proposition that minority stress increases risk for difficulties such as NSSI because it exacerbates mediational processes known to be associated with poorer mental health. In our sample, experiences of discrimination and expectations of rejection (i.e., minority stress) were associated with increased NSSI, in part, because of their connection to perceived burdensomeness. Minority stress may enhance a sense of burdensomeness among sexual minorities by increasing feelings of shame, a risk factor for self-harm that often co-occurs with feelings of burdensomeness [27]. Similarly, feelings of burdensomeness may result from perceptions that important others experience increased hassle, stress, or stigma as a result of one’s sexual minority status [32]. The finding that burdensomeness partially mediated the relationship between minority stress and NSSI is also consistent with prior studies showing a similar pattern with suicidal ideation [33, 49].

Thwarted belongingness was not a significant mediator in the association between minority stress and NSSI. Researchers theorize that burdensomeness may play a relatively stronger role in risk for NSSI, or suicide, than thwarted belonging because the two constructs are strongly related as evidenced by moderate to high correlations (e.g., 33). Low belongingness may actually enhance perceptions of burdensomeness, increasing the effect of burdensomeness within statistical models [27, 49]. Additionally, some have argued that belonging may not be as salient a risk factor for sexual minority college students because college provides a range of opportunities for belonging along with access to supportive student organizations, communities, or campus offices dedicated to sexual minorities [25, 33, 49, 50]. More research is needed to examine the potential for differential influence of risk factors across developmental periods [51], which may help to tease apart the unique effects of belonging and burdensomeness.

In addition to explaining increased risk for NSSI, the current study examined the influence of NSSI on suicide. The model explained 45 % of the variance in suicide thoughts and behavior, finding that NSSI retained a direct effect on suicide as well as an indirect effect through acquired capability. These results are consistent with the interpersonal theory of suicide [26], which suggests that suicide attempts and deaths are more likely once a person has desensitized to the fear and pain associated with enacting lethal self-harm. Few studies have examined the role of acquired capability in suicide risk for sexual minorities; yet, it follows that individuals who are more likely to experience victimization, discrimination, or violence might have a higher level of acquired capability. In a study of sexual minority adults, Ploederl and colleagues [52] report that acquired capability had one of the strongest associations with suicide attempts in their cluster analysis, whereas a lack of social support had the strongest association with suicidal ideation. These findings, along with the current results, indicate that sexual minorities may experience increased risk for suicide if their experiences lead to increased acquired capability for self-harm. Similarly, NSSI may retain its unique effect on suicide because it is an intention, self-directed action that results in an actual injury; possible approximating a suicidal act. Future research should examine the unique contributions of NSSI and other experiences (e.g., violence exposure) in the acquired capability for suicide among sexual minority individuals. Collectively, the current data support the need to continue to monitor suicidal thoughts and behaviors among individuals who report NSSI, and to utilize evidence-based therapies designed to target NSSI (e.g., [53]) and suicide (e.g., [54]).

It is important to interpret the current findings in the context of the study limitations. First, the data are cross-sectional; thus, we were unable to assess temporal relationships or establish causality. The data were obtained from a convenience sample that also used snowball sampling, and this may increase selection bias, limiting generalizability. The fact that our participants were college students further restricts the generalizability of the findings. However, due to our focus on a minority group, our results may generalize to other marginalized social groups who also experience societal exclusion. The self-report nature of the data is another limitation as self-report data are subject to recall and reporting biases. The use of a behavioral check-list to assess NSSI in which frequencies of behaviors are summed for a score could lead to inflations of severity (e.g., 50 acts of self-battery vs. 20 acts of self-cutting). While our sample is comprised of a large number of sexual minority youth, the size was still too small to examine within-group differences for our hypotheses. Emerging research suggests that there are different patterns of risk and associations between sexual minority identities, minority stress variables, and suicide risk [5, 33]. Studies with larger samples of sexual minority individuals should be careful to examine differences between those identifying as bisexual, gay/lesbian, questioning, or queer to determine whether unique patterns of risk exist within these subgroups. However, a key strength of our study is the inclusion of a greater diversity of sexual minority identities than has been represented in previous research. Lastly, the current study is one of only a few studies that tested a theoretically informed model to explain the elevated rates of NSSI within sexual minority youth, which offers greater direction for preventative intervention than descriptive studies.

The current findings suggest that a meaningful process exists whereby sexual minorities who experience minority stress may be at elevated risk for NSSI, especially if they also perceive themselves to be a burden on others. Subsequently, having engaged in NSSI appears to confer increased risk for suicidal thoughts and behaviors both directly and by increasing acquired capability. Efforts to treat and prevent NSSI and/or suicide among sexual minorities may begin by focusing interventions that reduce perceptions of burdensomeness and minority stress. Clinicians likely have the largest potential to reduce perceived burdensomeness by drawing upon cognitive (e.g., [55]) or relational oriented (e.g., [54]) therapies. Therapies that focus on strengthening coping and interpersonal skills (e.g., [56]) may help individuals manage minority stress, whereas mindfulness-based interventions might mitigate some effects of acquired capability. A key prevention strategy is to address societal and individual-level factors contributing to minority stress from a public health approach, working towards stigma reduction and public advocacy for policies that are supportive of sexual minorities [13, 46]. The current study expanded our understanding of NSSI within sexual minority youth by testing a theoretical model of risk. The field needs to continue to examine and compare theoretical models that can enhance our understanding of why NSSI and suicide are disproportionately experienced by sexual minority youth.
